# The oxysterol 27-hydroxycholesterol increases β-amyloid and oxidative stress in retinal pigment epithelial cells

**DOI:** 10.1186/1471-2415-10-22

**Published:** 2010-09-13

**Authors:** Bhanu Dasari, Jaya RP Prasanthi, Gurdeep Marwarha, Brij B Singh, Othman Ghribi

**Affiliations:** 1 Department of Pharmacology, Physiology and Therapeutics, University of North Dakota School of Medicine and Health Sciences, 501 North Columbia Road, Grand Forks, North Dakota, 58202, USA; 2 Department of Biochemistry and Molecular Biology, University of North Dakota School of Medicine and Health Sciences, 501 North Columbia Road, Grand Forks, North Dakota 58202, USA

## Abstract

**Background:**

Alzheimer's disease (AD) and age-related macular degeneration (AMD) share several pathological features including β-amyloid (Aβ) peptide accumulation, oxidative damage, and cell death. The causes of AD and AMD are not known but several studies suggest disturbances in cholesterol metabolism as a culprit of these diseases. We have recently shown that the cholesterol oxidation metabolite 27-hydroxycholesterol (27-OHC) causes AD-like pathology in human neuroblastoma SH-SY5Y cells and in organotypic hippocampal slices. However, the extent to which and the mechanisms by which 27-OHC may also cause pathological hallmarks related to AMD are ill-defined. In this study, the effects of 27-OHC on AMD-related pathology were determined in ARPE-19 cells. These cells have structural and functional properties relevant to retinal pigmented epithelial cells, a target in the course of AMD.

**Methods:**

ARPE-19 cells were treated with 0, 10 or 25 μM 27-OHC for 24 hours. Levels of Aβ peptide, mitochondrial and endoplasmic reticulum (ER) stress markers, Ca^2+ ^homeostasis, glutathione depletion, reactive oxygen species (ROS) generation, inflammation and cell death were assessed using ELISA, Western blot, immunocytochemistry, and specific assays.

**Results:**

27-OHC dose-dependently increased Aβ peptide production, increased levels of ER stress specific markers caspase 12 and gadd153 (also called CHOP), reduced mitochondrial membrane potential, triggered Ca^2+ ^dyshomeostasis, increased levels of the nuclear factor κB (NFκB) and heme-oxygenase 1 (HO-1), two proteins activated by oxidative stress. Additionally, 27-OHC caused glutathione depletion, ROS generation, inflammation and apoptotic-mediated cell death.

**Conclusions:**

The cholesterol metabolite 27-OHC is toxic to RPE cells. The deleterious effects of this oxysterol ranged from Aβ accumulation to oxidative cell damage. Our results suggest that high levels of 27-OHC may represent a common pathogenic factor for both AMD and AD.

## Background

Age-related macular degeneration (AMD) is the most common cause of irreversible vision loss in elderly population [1]. This disease is characterized by a progressive cell damage that targets the choroid, retinal pigment epithelium (RPE) and retina. Accumulation of drusen in the extracellular compartment between the choroid and the RPE is an early event in the course of AMD [2]. Drusen are composed of acute phase proteins, complement components, apolipoproteins, lipids, polysaccharides along with various other molecules [3-5]. Intriguingly, AMD has many pathological features that are common to Alzheimer's disease (AD), including the deposition of β-amyloid (Aβ) peptide [6]. Aβ is suggested to play a key role in AD pathogenesis by triggering oxidative stress, inflammation and cell death [7]. Aβ accumulation has also been demonstrated to be associated with drusen in eyes from AMD patients [8-10], mice models for AMD [11] and in RPE cells [12]. Recent studies from our laboratory have shown that the oxysterol 27-hydroxycholesterol (27-OHC) causes AD-like pathology by increasing Aβ production and triggers apoptotic cell death in human neuroblastoma SH-SY5Y cells [13,14] and in organotypic slices from rabbit hippocampus [15,16]. However, the extent to which and the mechanisms by which 27-OHC may also cause Aβ accumulation and cell death in *in vitro *model that is relevant to retinal pigment epithelial cells and AMD studies are lacking.

Similar to AD, the causes of AMD are not fully understood. Several lines of evidence suggest that genetic predisposition and environmental as well as dietary factors may contribute to the pathogenesis of these two progressive degenerative disorders. Recent epidemiological studies have demonstrated that high plasma cholesterol levels are associated with a high risk for AD [17]. Likewise, high intake of cholesterol and saturated fat is associated with increased AMD [18]. Cholesterol (free and esterified) is highly distributed in the human drusen [5,19,20]. The source of the cholesterol that accumulates in the retina is suggested to derive from both local cells and plasma origins [4,21-23]. Currently, the mechanisms by which cholesterol may increase the incidence of AD or AMD are not clear. Several lines of evidence suggest that oxidized cholesterol metabolites (oxysterols) may be the link by which cholesterol contributes to the pathogenesis of AD [24]. The oxysterol pathway has also been proposed as a unifying hypothesis for the cause of AMD [25-27].

Oxysterols are oxidation products of cholesterol that result from either autoxidation or enzymatic oxidation. While 7-ketocholesterol is the major oxysterol generated by autoxidation on the B hydrocarbon ring of cholesterol, 24-hydroxycholesterol, 25-hydroxycholesterol and 27-hydroxycholesterol are major oxysterols produced by enzymatic oxidation on the lateral chain of the cholesterol structure. Oxysterols have diverse physiological and biochemical functions ranging from regulation of cholesterol homeostasis to regulation of nuclear receptors [26-28]. However, abnormal oxysterol levels can cause oxidative stress, inflammation and apoptotic cell death [25,28-32].

In this study, we determined in the human RPE cell line ARPE-19 the effects of 27-OHC on pathological hallmarks that are common to both AMD and AD. We have specifically determined levels of Aβ and β-site of APP cleaving enzyme (BACE-1), which initiates Aβ production, caspase 12 and gadd153 as markers of ER stress, mitochondrial membrane potential as a marker of mitochondrial stress, the nuclear factor κB (NFκB) and heme-oxygenase 1 (HO-1) two proteins activated by oxidative stress, ROS generation and glutathione depletion and TNF-α as marker of inflammation. Additionally, calcium homeostasis, cytotoxicity and cell death assays were also carried out.

## Methods

### Cells and Treatments

ARPE-19 cells (American Type Culture Collection, Manassas, VA) were grown in DMEM/F12 Glutamax media with 10% FBS and standard antibiotics (100 IU/mL penicillin, and 100 μg/mL streptomycin, Sigma, St. Louis, MO) in a 5% CO_2_, 37°C incubator. Cells were seeded in 75-cm^2 ^flasks, 6-well plates or 96-well plates. All cell culture reagents were obtained from Invitrogen, Carlsbad, CA. 27-OHC was obtained from Medical Isotopes Inc, Pelham, NH. Stock solutions of 27-OHC were prepared in 100% ethanol and stored at -70°C. Working concentrations were prepared by dissolving 27-OHC stock solution in media. When cells reached confluency, they were incubated with 0, 10 or 25 μM 27-OHC in culture media for 24 hrs, time by which 27-OHC increases Aβ levels in SH-SY5Y cells [13]. Experiments were carried out in triplicate. The concentrations of the 27-OHC used in the present study are the same as those we demonstrated to cause AD-like pathology in human neuroblastoma cells and in organotypic slices [13,15]. Joffre and colleagues have used concentrations up to 50 μM of 7-ketocholesterol, 24-hydroxycholesterol, and 25-hydroxycholesterol [30].

### Quantification of Aβ by Enzyme-Linked Immunosorbent Assay (ELISA)

Cells were treated with 27-OHC for 24 hrs. Spent media was collected, protease and phosphatase inhibitors cocktail (Thermo Scientific, Rockford, IL) was added. The media was centrifuged at 16,000 × *g *for 5 min at 4°C. 100 μL of supernatant was used for Aβ1-42 and Aβ1-40 quantification by colorimetric sandwich ELISA (Covance, Denver, PA) according to the manufacturer's instructions and as we have previously described [13]. Aβ1-42 and Aβ1-40 levels were expressed in pg/mL.

### Western Blot Analysis

After 24 hrs treatment with 27-OHC, cells were harvested on ice and homogenized in a mammalian protein extraction reagent (M-PER, Thermo Scientific, Rockford, IL) supplemented with protease and phosphatase inhibitors. Nuclear extracts for NFκB were prepared by using NE-PER extraction kit (Thermo Scientific, Rockford, IL). Protein concentrations were determined with bicinchoninic acid (BCA) protein assay reagent. Proteins (10 μg) were separated on SDS-PAGE gels followed by transfer onto a polyvinylidene difluoride (PVDF) membrane (Biorad, Herculus, CA) and incubation with antibodies to BACE-1 (Mouse, 1:1000, Millipore, Bedford, MD), caspase 12 (Rat, 1:1000, Abcam, Cambridge, MA), gadd153 (Mouse, 1:1000, Abcam, Cambridge, MA), and HO-1 (Mouse, 1:500, Assay Designs, Ann Arbor, MI). β-actin (Mouse, 1:5000, Santa Cruz Biotechnology, Santa Cruz, CA) was used as a gel loading control. 5 μg of nuclear extracts were used for NFκB (NFκB p65; Mouse, 1:100, Santa Cruz Biotechnology, Santa Cruz, CA) and lamin (Rabbit, 1:500, Cell Signaling Technology, Inc. Danvers, MA) was used as loading control. For antibodies of mouse origin, goat anti-mouse secondary antibody conjugated with horseradish peroxidise (HRP) was used (1:5000, Biorad, Herculus, CA). For caspase12, goat anti-rat secondary was used (1:2500, Santa Cruz Biotechnology) and for lamin, goat anti-rabbit secondary antibody (1:5000, Biorad) was applied. The blots were developed with enhanced chemiluminescence (Immmun-star HRP chemiluminescent kit, Bio-Rad, Herculus, CA). Bands were visualized on a PVDF membrane and analyzed by LabWorks 4.5 software on Ultra Violet Products (UVP) Bioimaging System (Upland, CA). Results were quantified by densitometry normalized to β-actin or lamin and analyzed as total integrated densitometric values (arbitrary units).

### Confocal Microscopy

ARPE-19 cells were grown on collagen coated coverslips and treated with 27-OHC for 24 hrs. After incubation cells were washed with PBS, fixed in 4% paraformaldehyde, blocked with 5% normal goat serum and incubated overnight at 4°C with monoclonal mouse antibodies to gadd153 (1:250, Abcam, Cambridge, MA) or NFκB p65 (1:250, Santa Cruz Biotechnology, Santa Cruz, CA). Cells were washed in PBS, incubated with secondary antibody conjugated to Alexa fluor-488 (Molecular Probes, Inc., Eugene, OR) for 1 hour at room temperature, washed with PBS, mounted with Vectashield containing DAPI (Vector Laboratories Inc, Burlingame, CA), and visualized with a Zeiss LSM 510 META confocal system coupled to a Zeiss Axiophot 200 inverted epifluorescence microscope. Imaging was performed with a 63X oil immersion objective.

### Calcium Imaging

Cells were cultured on 35 mm glass bottom culture dishes (MatTek Corp, Ashland, MA) and incubated with 0, 10 or 25 μM of 27-OHC for 24 hrs. To measure intracellular Ca^2+^, cells were loaded with 2 μM fura-2AM (Calbiochem, La Jolla, CA) for 45 min at 37°C under an atmosphere of 5% CO_2_-95% air, washed three times with Ca^2+^-free SES buffer [33]. For fluorescence measurements, the fluorescence intensity of Fura-2AM -loaded cells were monitored using a CCD camera-based imaging system (Compix Inc, Cranberry, PA) mounted on an Olympus XL70 inverted microscope equipped with an Olympus 40× (1.3 NA) fluor objective. A monochrometer dual wavelength enabled alternative excitation at 340 and 380 nm, whereas the emission fluorescence was monitored at 510 nm with an Orka imaging camera (Hamamatsu). The images of multiple cells collected at each excitation wavelength were processed using the Ca^2+ ^imaging, PCI software (Compix Inc., Cranbery, PA) to provide ratios of Fura-2 fluorescence from excitation at 340 nm to that of excitation at 380 nm (*F*_340_/*F*_380_).

### Glutathione Depletion, ROS Production, Mitochondrial Membrane Potential, TNF-α and Cytotoxicity Assays

Equal numbers of cells were seeded in 96-well plates. Cells were treated with 27-OHC for 24 hrs and media was collected. A luminescent based GSH-Glo Assay (Promega, Madison, WI) was used for quantification of glutathione in cells. The intracellular production of ROS was measured using Dichlorofluorescein diacetate (DCFH-DA) assay (Sigma, St. Louis, MO). JC-1 Mitochondrial Membrane Potential Detection kit was used to measure mitochondrial membrane potential changes in cells (Biotium, Hayward, CA). The Invitrogen Mouse Tumor Necrosis Factor-α (Ms TNF-α) ELISA was used for the quantitative determination of TNF-α. Cytotoxicity assay was performed using Cyto Tox-ONE Homogenous membrane integrity assay (Promega). All the assays were performed according to the manufacturer's instructions.

### TUNEL Assay

The DeadEnd™ Fluorometric TUNEL System (Promega) was used for detection of apoptotic cells. ARPE-19 cells were fixed with 4% paraformaldehyde at room temperature, washed with PBS, permeabilized with 0.1% Triton X-100, and labelled according to manufacturer's instructions.

### Statistical analysis

Data was analyzed for statistical significance using analysis of variance (ANOVA) followed by Dunnett Multiple Comparison Test with GraphPad Prism software 4.01. All values obtained from the three different experiments were expressed as mean value ± SEM. Comparisons were considered significant at p < 0.05.

## Results

### 27-OHC increased Aβ levels

**Figure 1:**
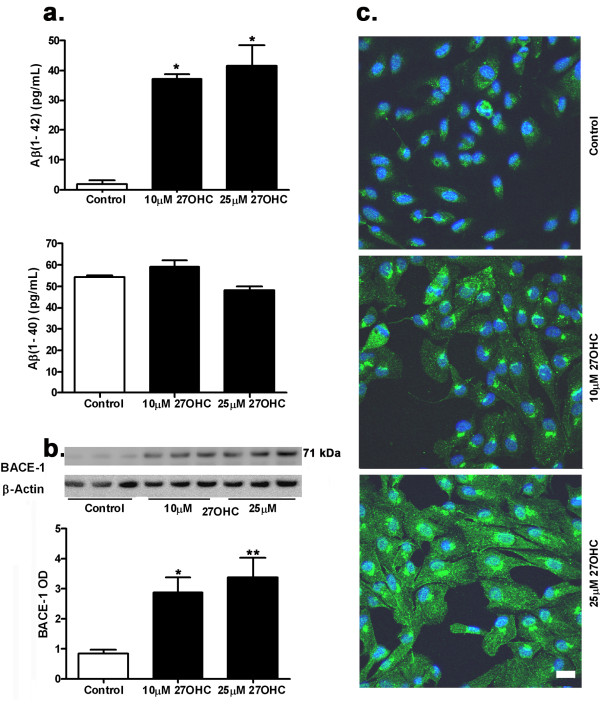
**27-OHC increased Aβ production**. Treatment of ARPE-19 cells with 10 μM and 25 μM 27-OHC for 24 hrs increased Aβ1-42 but not Aβ1-40 levels (a). Levels of BACE-1, the enzyme that initiates the generation of Aβ, are also increased following treatment with 27-OHC (b). Immunocytochemistry with 6E10, an antibody that detects Aβ, shows increased staining with 27-OHC (green); DAPI (blue) was used as a nuclear counterstain (c). *p < 0.05, **p < 0.01 vs control; Bar, 20 μm.

Aβ is an important component of plaques in Alzheimer's disease and drusen deposits in AMD. Aβ1-42 and Aβ1-40 are the two major peptides that increase in AD. The ELISA assay for Aβ quantification (Figure 1a) showed that the ARPE-19 cells express basal levels of Aβ1-42 and Aβ1-40. Treatment with 27-OHC at 10 and 25 μM for 24 hrs significantly increased Aβ1-42 but not Aβ1-40 levels in these cells. The magnitude of increase in Aβ1-42 levels is similar with 10 μM and 25 μM of 27-OHC. Our results showed that 27-OHC also increased the levels of BACE-1 enzyme (Figure 1b), suggesting that the increased levels of Aβ1-42 derive, at least in part, from an increase in the rate of production of this peptide through the processing of β-amyloid precursor protein by BACE-1.

The immunocytochemistry using 6E10 antibody that detects Aβ peptides also showed an increase in the Aβ immunoreactivity with both 10 μM and 25 μM of 27-OHC compared to untreated cells (Figure 1c). The Aβ staining appeared to be mostly perinuclear. The immunocytochemistry results confirmed the ELISA results showing increased Aβ1-42 levels. Increased Aβ1-42 peptide levels in the ARPE-19 cells could cause oxidative damage to these cells, as high levels of this peptide either soluble or insoluble form is toxic to cells.

### 27-OHC disturbed Ca^2+ ^homeostasis and caused ER stress

**Figure 2:**
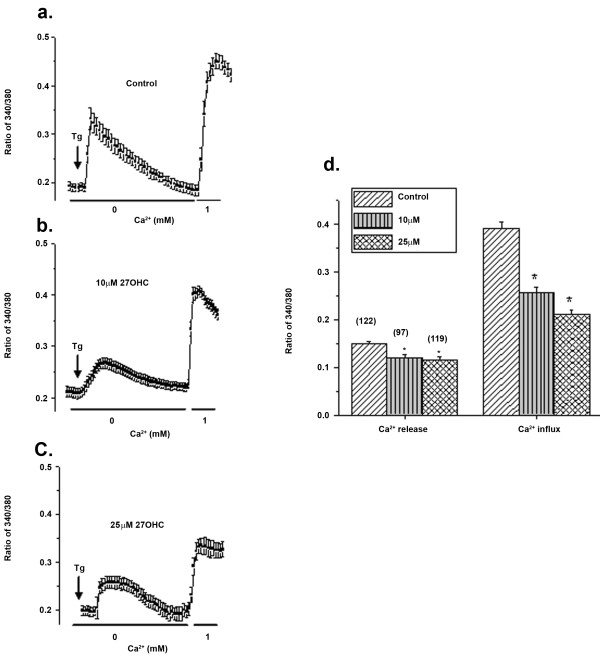
**Ca^2+ ^homeostasis is disturbed by 27-OHC**. Ca^2+ ^imaging was performed in the presence of thapsigargin (Tg; 2 μM in a Ca^2+^-free media) in control ARPE-19 cells (a) or cells treated with 10 μM (b) or 25 μM 27-OHC (c). Ca^2+ ^influx was measured by the addition of 1 mM Ca^2+ ^externally and traces shown here are averages of 30-40 cells in each condition (d). Bar graph indicates the mean values of the first peak (ER calcium release) and second peak (calcium entry). *p < 0.05 vs control.

Ca^2+ ^is essential for cell functioning, and cell survival depends on the maintenance of Ca^2+ ^homeostasis [34]. Disruptions of Ca^2+ ^homeostasis result in the development of the ER stress response which can compromise cell survival. Our results showed that addition of thapsigargin (Tg, 2 μM), which initiates release of Ca^2+ ^from the internal ER stores (in Ca^2+ ^free media) showed a significant decrease in ER Ca^2+ ^content in cells treated with either 10 or 25 μM of 27-OHC (Figure 2a-c). Importantly, addition of 1mM Ca^2+ ^externally showed a robust increase in cytosolic Ca^2+ ^levels in control cells. However, cells treated with 10 or 25 μM 27-OHC showed a gradual decrease in Ca^2+ ^entry (40-50% decrease). Mean Ca^2+ ^influxes from 90-120 individual cells are shown as bar graph in Figure 2d. Basal Ca^2+ ^influx (measured upon addition of external Ca^2+ ^without Tg stimulation) was not altered (data not shown). Overall, these data demonstrate that treatment of the cells with 27-OHC leads to a decrease in intracellular Ca^2+ ^entry and thus decreased ER Ca^2+^. This could lead to ER stress, since Ca^2+ ^entry through the plasma membrane is essential for the refilling of the ER store.

**Figure 3:**
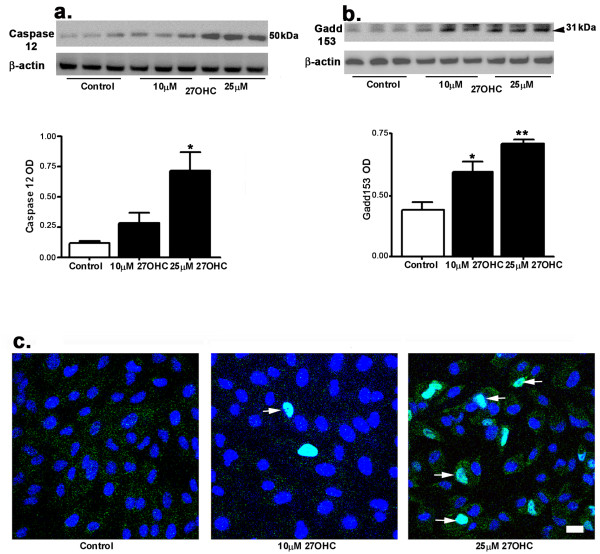
**27-OHC activated ER stress**. Western blots showing an increase in levels of caspase 12 (a) and gadd153 (b), two specific markers of ER stress. (c) Immunocytochemistry shows that gadd153 immunorectivity (green) is localized in nucleus (DAPI, blue) following treatment with 27-OHC (arrows). *p < 0.05, **p < 0.01 vs control; Bar, 20 μm.

As a result of ER stress, caspase 12 is activated and can lead to apoptotic cell death [35]. Our Western blot results showed that 27-OHC treatment increased the levels of caspase 12, a higher increase at 25 μM than at 10 μM (Figure 3a). Sustained ER stress leads to activation of gadd153 which can cause cell-cycle arrest and/or apoptosis. Our results show that 27-OHC increased levels of gadd153 at both 10 and 25 μM concentrations (Figure 3b). The confocal microscopy imaging revealed an increase in the number of nuclei that are gadd153-positive on treatment with 25 μM 27-OHC treatment (Figure 3c, arrows). The immunocytochemistry results confirmed the activation of gadd153 and its translocation into the nucleus.

**Figure 4:**
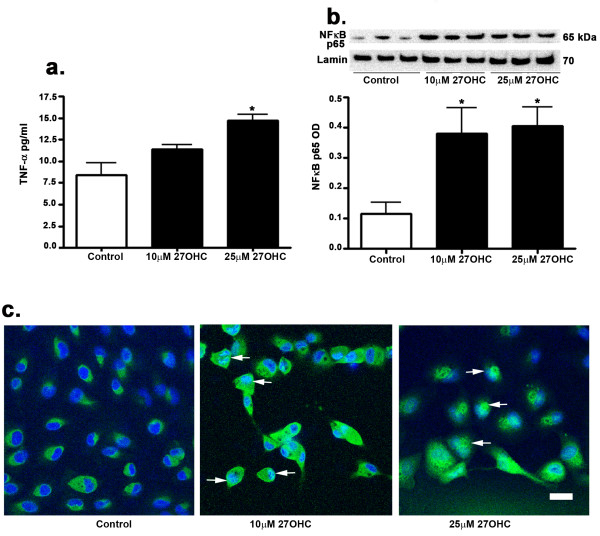
**Effects of 27-OHC on TNF-α levels and NFκB**. Treatment with 27-OHC increases TNF-α levels, as shown by ELISA (a) and increased NFκB levels in the nucleus as shown by Western blot (b). Immunocytochemistry for NFκB shows increased immunoreactivity (green) and translocation into the nucleus (DAPI, blue) of this protein in the 27-OHC-treated cells (arrows, c). *p < 0.05 vs control; Bar, 20 μm.

TNF-α is a multifunctional pro-inflammatory cytokine that is viewed as a classic regulator of cell death and is also activated by ER stress [36]. TNF-α is also an activator of NF-κB. Our results show that 27-OHC, at 25 μM but not at 10 μM, induces an increase in TNF-α levels as determined by an ELISA assay (Figure 4a). Our results suggest that treatment with 27-OHC is associated with inflammatory responses.

NFκB can be activated by ER stress and can exert either cytoprotective or cytotoxic effects depending on the type of stimulus and duration [37]. Active NFκB is known to translocate to the nucleus [38]. Here we showed that NFκB levels in the nucleus are increased in the 27-OHC treated cells (Figure 4b). The immunocytochemistry with antibody to NFκB further showed an emergence of nuclear staining for NFκB with 27-OHC treatment (Figure 4c, arrows).

### 27-OHC-induced oxidative damage

**Figure 5:**
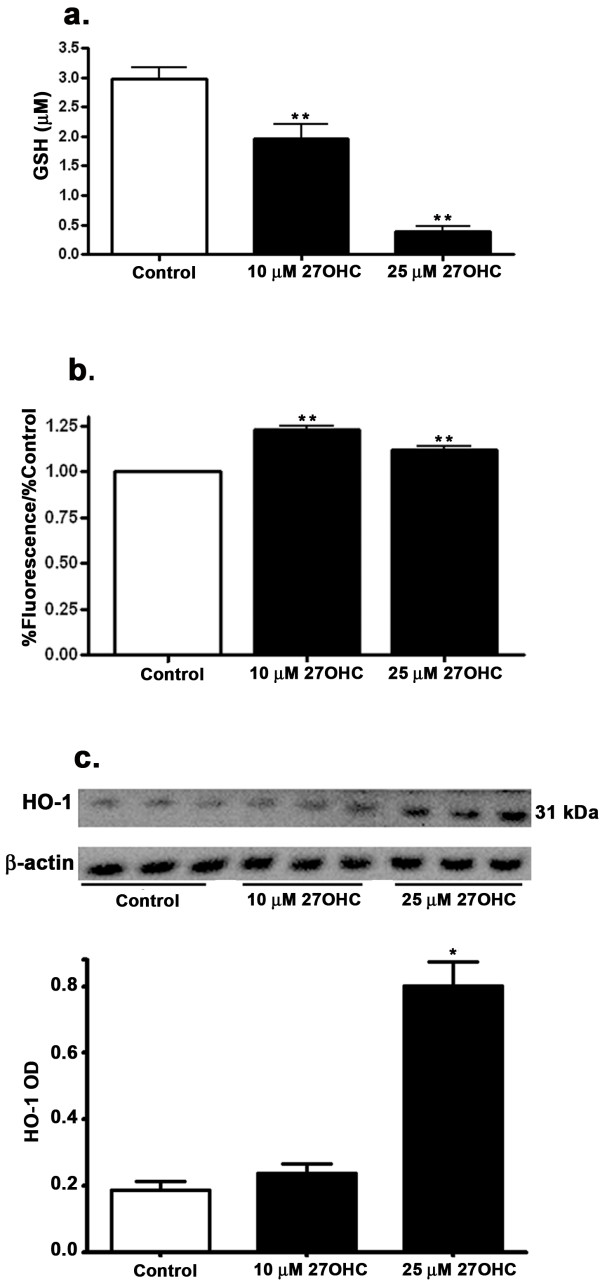
**27-OHC effects on glutathione, ROS and HO-1**. Treatment with 27-OHC causes a decrease in glutathione concentrations (a), an increase in ROS generation as measured by DCFH-DA assay (b) and an increase in HO-1 levels (c). *p < 0.05, **p < 0.01 vs control.

The glutathione system is the main redox control system of the cell. It includes reduced glutathione (GSH) and oxidized glutathione (GSSG). Treatment with 10 μM or 25 μM 27-OHC caused a significant reduction in GSH levels (Figure 5a). These results suggest that 27-OHC reduced the anti-oxidant potential of cells, thereby increasing cell susceptibility to oxidative damage. We also determined the extent to which treatment with 27-OHC caused oxidative stress in ARPE-19 cells by increasing the generation of ROS. The nonfluorescent dichlorofluorescin (DCFH), upon oxidation, is converted to DCF and emits fluorescence. Because DCFH can be oxidized by various ROS, the increase of intracellular DCF fluorescence therefore reflects an overall oxygen species index in cells. We have found that 27-OHC increased DCFH fluorescence, indicating that treatment with this oxysterol can generate ROS in ARPE-19 cells (Figure 5b). Excessive production of ROS is known to lead to oxidative stress, loss of cell function, and ultimately to cell death. Treatment of cells with 25 μM 27-OHC but not 10 μM concentration increased HO-1 (Figure 5c).

### 27-OHC induced cell death

**Figure 6:**
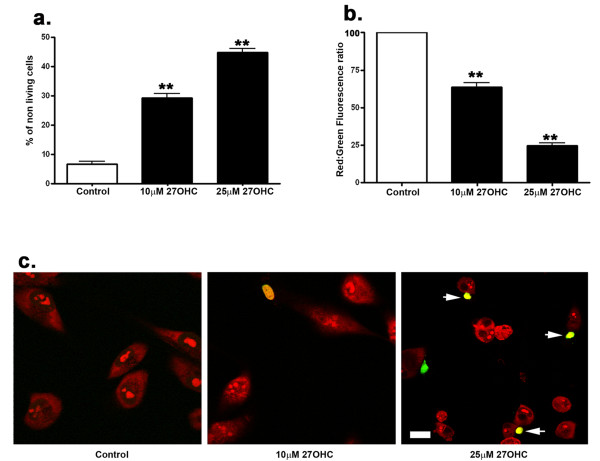
**27-OHC is deleterious to ARPE-19 cells**. A Cyto Tox-ONE Homogenous membrane integrity assay shows that 27-OHC is cytotoxic to cells (a) and decreases the mitochondrial membrane potential as measured by a reduction in red (non-apoptotic cells)/green (apoptotic cells) fluorescence (b). TUNEL assay shows that 27-OHC increases the number of apoptotic cells (arrow) with 25 μM 27-OHC (c). Apoptosis is evidenced by DNA fragmentation labeled with fluorescein (green); Propidium iodide (red) is used to label cell nuclei. **p < 0.01 vs control, Bar, 20 μm.

After 24 hrs treatment, the effect of 27-OHC on cell viability was quantitatively determined by the measurement of lactate dehydrogenase (LDH) released from cells into the medium by using the CytoTox-ONE™ Homogeneous Membrane Integrity Assay. LDH is released into the surrounding medium from cells that have lost membrane integrity. This fluorometric assay estimates the number of nonviable cells by measuring the release of LDH from cells with a damaged membrane. The amount of fluorescence produced is proportional to the number of lysed cells. Our results showed that 27-OHC treatment leads to a significant increase in the number of dead cells (Figure 6a). Cell death involves various events including the loss of mitochondrial membrane potential [39]. The fluorescent cationic dye JC-1 (5, 5', 6, 6'-tetrachloro-1,1',3,3' tetraethylbenzimidazolyl-carbocyanine iodide) was used in our experiments that signals the loss of mitochondrial membrane potential [40]. In apoptotic cells, JC-1 exists in the green fluorescent (529 nm) monomeric form because of decreased mitochondrial membrane potential. In non-apoptotic cells, JC-1 accumulates as aggregates in the mitochondrial membranes, resulting in red fluorescence (590 nm) [41]. The ratio of red to green fluorescence was determined. Our results show that 10 μM and 25 μM 27-OHC reduces the red: green ratio, indicating a decrease in the mitochondrial membrane potential (Figure 6b). TUNEL assay, used to detect apoptotic cell death, showed no TUNEL-positive cells in untreated cells; in the 27-OHC-treated cells, TUNEL-positive cells were observed with a number that is higher with 25 μM than 10 μM 27-OHC (Figure 6c). All together, these results demonstrate that 27-OHC compromises cell survival.

## Discussion

Epidemiological studies show that increased plasma cholesterol levels are associated with atherosclerosis and increased risk of dementia, including AD [17,42]. Although, there is no consensus on the association of plasma lipid levels and AMD, two recent genome wide association studies implicated cholesterol metabolism involvement in AMD [43,44]. Drusen on Bruch's membranes in AMD exhibit increased deposition of lipids, including cholesterol. The origin of lipids that accumulate in these structures has been a source of debate, with studies suggesting intraocular source and/or plasma contribution to lipid accumulation in the eye (see for review [45]). It is also possible that cholesterol oxidized metabolites (oxysterols) in plasma can gain access to the eye. Similar access of plasma oxysterols to the brain has been reported [46]. It is also possible that locally made cholesterol in the eye is associated with increased levels of oxysterols in certain conditions. As an example, sterol 27-hydroxylase (CYP27A1)-the enzyme that converts cholesterol to 27-OHC has been shown to localize in retina [22,47]. It is known that deficiency in 7-α-hydroxylase, an enzyme needed to prevent 27-OHC accumulation, causes severe neonatal cholestatic liver disease, where 27-OHC concentrations in the plasma could reach up to 800 μM [48]. Similar abnormal accumulation of these metabolites in the retina may put this tissue in danger of degeneration. Of these oxysterols, 7-ketocholesterol has been suggested to play a key role in the pathogenesis of AMD [25] and has been recently shown to induce DNA damage in human RPE cells [49]. Other oxysterols such as 24-OHC, 25-OHC, 27-OHC, and 7β-OHC triggered inflammation, induced oxidative stress and apoptotic cell death [32,50-53] in various cells. However, further studies are warranted to measure oxysterols in the eyes of hypercholesterolemic animals or humans.

Mounting evidence demonstrates that oxysterols have deleterious effects that may contribute to the pathogenesis of AD and AMD. However, the intracellular mechanisms underlying oxysterols toxicity are not well known. A common pathological feature that accumulates both in AD and AMD is Aβ [8,9], a peptide that is considered to play a central role in the neurodegenerative processes by increasing oxidative stress and cell damage. We have recently shown that 27-OHC, but not 24-OHC, increases Aβ peptide levels in human neuroblastoma cells [13] and in rabbit hippocampus [15]. In the present study, we demonstrate for the first time that 27-OHC also increases the levels of Aβ in retinal pigment epithelium ARPE-19 cells. Aβ deposition could be an important element in the local inflammatory and oxidative processes that contribute to the deterioration of photoreceptors and pathogenesis of AMD. The β-amyloid precursor protein is present in the RPE cytoplasm and Aβ-labeled structures were also identified in RPE cells [9], suggesting that RPE cells have the ability to generate Aβ. Our results show that Aβ accumulation at least in part is due to increased generation of this peptide by the action of BACE-1, the enzyme that initiates the cleavage of β-amyloid precursor protein.

Accumulation of Aβ and oxidative damage are intrinsically related. Oxidative damage in cells can be caused by a variety of factors including ER stress, ROS generation, glutathione depletion and inflammation. ER stress has been implicated in both AD (see for review [54]) and AMD (see for review [55]). The ER is the cell compartment where various proteins are synthesized and Ca^2+ ^is stored. Cellular stress leads to depletion of ER Ca^2+ ^stores, activation of specific ER stress proteins such as caspase 12, gadd153 and apoptotic cell death [56]. Our results show that 27-OHC-induced ER stress may be due to the loss of Ca^2+ ^influx and a decrease in ER Ca^2+ ^levels. This was consistent since 27-OHC showed activation of the ER specific apoptotic proteins caspase 12 and gadd153. Gadd153 is present in the cytosol under normal conditions and translocates to the nucleus following sustained ER stress [57]. Induction of gadd153 was shown to upset the cellular redox state by depleting cellular glutathione and exaggerating the production of ROS [58]. We also showed that the 27-OHC-induced gadd153 activation is accompanied by increased levels of ROS and depleted glutathione.

HO-1 is mainly located in the ER and stimulates the oxidation of cholesterol to oxysterols. It is regarded as a sensitive marker of oxidative stress in cells and tissues [59,60]. Its induction is suggested to be an early event in the pathogenesis of sporadic AD [61]. HO-1 levels were also increased in RPE of AMD-affected maculas [62]. HO-1 gene expression is stimulated by ER stress [63]. ER stress can also cause apoptosis via NFκB activation [64]. In a basal state, NFκB is sequestered in the cytoplasm by IκB protein. ER stress and ROS degrade IκB, leading NFκB translocation to the nucleus where NFκB induces transcription of target genes. Activation of NFκB was observed in AD [65] as well as in AMD [66]. Our results showed that 27-OHC increases levels of HO-1 and activated NFκB. This suggests the potential role of 27-OHC as a pro-oxidant that can cause oxidative damage to retinal cells and in the pathophysiology of AMD.

Increased levels of Aβ, ER stress, HO-1 and NFκB are accompanied by glutathione depletion as well as elevated levels of ROS. These results demonstrate the potential oxidative effects of 27-OHC. Increased levels of TNF-α suggest that 27-OHC-induced oxidative stress is associated with triggering of inflammatory processes. All together, oxidative stress and inflammation may ultimately lead to cell death. Our study further demonstrates that 27-OHC is cytotoxic to cells, induces the loss of mitochondrial membrane potential and causes apoptotic cell death as detected by the TUNEL assay.

While it provides important information on the deleterious effects of 27-OHC effects in retinal pigment epithelial, this study has some limitations. For example, the extent to which the effects we show here are limited to 27-OHC remains to be investigated as the effects of other structurally similar oxysterols, such as 24-OHC, 25-OHC or 27-hydroxy-7-ketocholesterol have not been determined. Additionally, the concentrations of 27-OHC and other oxysterols are not known in healthy or diseased retinas. The concentrations used for 27-OHC here were based on concentrations cited in literature for various oxysterols as well as from our recent studies in neuroblastoma cells and organotypic slices. Oxysterol internalization assessment, which would allow an assessment of the actual concentrations that reach the cytoplasm were not carried out. Future studies comparing the effects of 27-OHC to other oxysterols and assessing their internalization are warranted in order to determine whether the effects we observed are physiological and are specific to 27-OHC.

## Conclusions

In summary, our study demonstrates that 27-OHC is deleterious to retinal pigment epithelial cells by triggering the accumulation of Aβ peptide, ER stress and oxidative damage, which are all pathological hallmarks of both AD and AMD. Our data suggests that 27-OHC may be a common factor that contributes to the pathogenesis of both AD and AMD. Preventing excess oxidation of cholesterol to 27-OHC and/or reducing the accumulation of 27-OHC would probably preclude the risk of oxidative damage to brain and retinal epithelial cells.

## Competing interests

The authors declare that they have no competing interests.

## Authors' contributions

BD did most experiments and analyzed the data. ELISA and ROS experiments were done by JPRP. GM advised in design and participated in Western blot experiments. BBS conducted calcium studies. OG conceived the study and supervised the results and wrote the final draft. All authors read and approved the final manuscript.

## Pre-publication history

The pre-publication history for this paper can be accessed here:

http://www.biomedcentral.com/1471-2415/10/22/prepub
